# Dictating play to the left wing: Does soccer make you more Democratic?

**DOI:** 10.3389/fspor.2023.1004695

**Published:** 2023-03-23

**Authors:** Johan M. Rewilak

**Affiliations:** Department of Sport and Entertainment Management, University of South Carolina, Columbia, SC, United States

**Keywords:** political ideology, Major League Soccer, FIFA world cup, women’s world cup, Democratic Party, Republican Party

## Abstract

There is a correlation between soccer's popularity and states that traditionally vote Democrat in US elections. This has led to claims that where democrats lead, soccer follows. Yet, this relationship may not be entirely stable, as soccer may stimulate the Democratic party vote through its multicultural elements. Using the 1994 World Cup as a plausibly exogenous shock that positioned US soccer, we investigate whether US states that hosted the tournament increased their Democratic vote in future Presidential elections. A two-way fixed-effects estimator and a dynamic difference-in-difference estimator shows that if a US state was a 1994 World Cup host, it increased its Democratic vote share. However, when examining Major League Soccer franchises, this relationship breaks down but recovers when investigating the women's World Cup in 1999 and 2003. As the swing states of Florida and Georgia are hosting 2026 World Cup matches, the findings may hold key insights for the 2028 Presidential election.

## 1. Introduction

The political landscape in the United States is heavily polarised, with two dominant parties, the Democratic Party and the Republican Party (1, 2). The Democratic Party is claimed to have members who are more liberal and open-minded with their views, and in comparison, Republican voters are more traditional and conservative in their beliefs (3). An examination of the political spectrum reveals that the Democratic Party sits to the left of the Republican Party, although this does not imply that the Democrats position themselves on the extreme left of the spectrum and Republicans on the far right (3).

There are many characteristics such as race, age, geography, and other socioeconomic characteristics that explain US voting behaviour (4–7). For example, many rural areas in the mid-West of America are Republican strongholds, with urban areas tending to vote Democrat (8).

Similarly, the United States exhibits a wide divide in soccer participation and popularity, primarily fuelled by geography (9).^1^ This divide may be political, where soccer is most popular in blue states and least popular in red states (10). In addition, soccer fans are viewed as “smug, liberal, elites” in the United States (11), which is consistent with the idea that the game was shaped by social elites from England's public schools (12). In addition, Woods (13) empirically found a significant negative correlation between areas that voted Republican and supported soccer.

Therefore, is there a further divide between US voters, the soccer-loving Democrats, and the soccer-objecting Republicans? Hence, can sports polarise voters, just like other socio-demographic characteristics, and are soccer fans more left wing in their political ideology?

Despite England's social elites shaping the game of soccer (12), the sport is viewed as a working-class one. This is because 5 of the founding 12 clubs of the English football league came from Northern mill towns (14). The game flourished in these areas, as industrial workers, upon receiving Saturday afternoons off work in the 1850s, would congregate to watch or play the sport (15). Despite the decline in class-led voting, historically, the working-classes would vote for the more left-leaning party in the United Kingdom.

Nevertheless, could this relationship be reversed: where soccer's presence makes a region more democratic? This is one reason that motivates this work, as it is possible that causation may run in both directions. It is equally possible that soccer fans become more democratic, as it is the Democrats who are more likely to turn to soccer. Given this, we test the former relationship that has been ignored in favour of just accepting the latter.

Theoretically, homophily—where people seek out or are attracted to individuals similar to themselves—may be one method how this may arise (16). Specifically, the mechanism could be via cluster migration (17), where migrants may be attracted to areas where soccer is popular, or where people have a strong interest in soccer to pursue opportunities to either play or watch the sport.

Social cohesion theory, where members of a community can build or strengthen existing relationships upon shared values (18), may also alter the political make-up of a region. The contact hypothesis (19) may outline how soccer's diverse popularity may stimulate the local electorate to become more culturally tolerant of one another. Furthermore, with increased interactions with liberal people, peer effects may further enhance the support of the Democratic Party. These are further channels of how soccer may fuel a higher liberal vote.

Therefore, we investigate whether US states that were exposed to a soccer-promoting shock tend to vote more democratically in US Presidential elections. To address this research question, the two-way fixed effects (TWFE) difference-in-difference (DID) estimator and the dynamic difference-in-difference estimator are used.

It is assumed that initial conditions matter, and that the Fédération Internationale de Football Association (FIFA) World Cup in 1994 acted as a plausibly exogenous shock that reset soccer in the United States. States that contained host cities had greater exposure to soccer than non-hosts, which may have influenced voting decisions in subsequent US Presidential elections. Host city selection is non-random, given the criteria required to be a viable World Cup host. Thus, the methodology requires a suitable comparator group to the treated host states. The 1994 bidding process outlined 18 cities in its initial application, of which only 5 were selected as final venues. In addition, four further venues were selected to host World Cup matches that were not initially considered. Therefore, from a total of 22 potential venues, 13 were not selected, providing a good counterfactual to the nine treated venues. This validates the use of this estimation strategy and shows that decision-makers had a significant choice to make when determining which venues would host the World Cup.

It is also unlikely that host city selection was a political decision based on future vote shares, given the alternative priorities the United States faced with its World Cup bid. Furthermore, by focusing on the state vote share, rather than the county level, any bias that cities tend to vote Democratic rather than Republican should be overcome (8). This helps validate the estimation strategy.

By hosting the 1994 World Cup, the United States committed to create a professional men's league. Major League Soccer (MLS) was founded in 1996, with approximately a dozen clubs from 1996 to 2004.^2^ Many of these clubs entered markets in states that held the FIFA World Cup in 1994 and hosted the women's 1999 and 2003 World Cups.^3^^,^^4^ Such a high overlap supports the idea of using the FIFA World Cup in 1994 as a plausibly exogenous shock to investigate the research question.

The findings show that states that hosted the men's World Cup increased their Democratic vote share by 0.16 points. When testing if this was attributable to MLS, this effect vanishes. Yet, when investigating the impact of the women's World Cup, held in 1999 and again in 2003, this positive impact recovers, and the point estimate is 0.15, similar to the men's magnitude.

When examining the heterogenous effects through time, the largest point estimate for the men's World Cup is in 2000. Similarly, the largest point estimate for the women's World Cup is also the treatment period 2000. However, in both cases, the value is not statistically different from the treatment effects in 1996 and 2004 for the men's World Cup and in 2004 for the women's tournament. When examining the joint impact from both men's and women's World Cups, the impact remains positive and statistically significant.

The evidence suggests that only international scale soccer events increased the Democrat vote in soccer hosting states. This is intuitive, as when mega sporting events occur, a host city is exposed to sports tourists from many different nationalities and cultures, unlike in a domestic competition.

The United States is co-hosting the 2026 World Cup, recently announcing its host cities. Half of these cities are in Republican states categorised by the Cook Partisan Voting Index (CPVI), and both Georgia and Florida are swing states. Whereas this paper does not attempt to predict or forecast future Presidential elections, if soccer does make you more democratic, then it is possible that the Democratic Party may gain some vital electoral college votes in these states. However, soccer does operate from a different popularity base in terms of both participation and viewership than that of 1994, which implies that caution should be exercised when making predictions about voting behaviour in the 2028 Presidential election.

Political polarisation has increased in the United States at the time of writing (20, 21). This may also impact soccer's influence on voting behaviour. The implications of this are that the median voter may no longer swing and just align themselves with one of the two main political parties (20). In terms of voting behaviour, it may be that they remain faithful to the party that is closest to their beliefs, given that the rival party may have moved too far away from their position, where this loyalty may persist until the parties converge once again on the political spectrum.

The rest of this manuscript is structured as follows: Section 2 provides a brief history of soccer in the United States and then summarises the relevant literature. Section 3 presents the data and methods, with Section 4 presenting the findings. Section 5 discusses the results and concludes the manuscript.

## 2. Background and existing literature

### 2.1. Brief history of US soccer

The immigrant population from Europe brought soccer to the United States, where it was played as early as the 1850s in Louisiana (22). Unlike other sports, soccer never established a strong foothold in the country, despite the United States entering and finishing third at the inaugural 1930 FIFA World Cup. In the 1960s, soccer resurged with the launch of the North American Soccer League (NASL).

The NASL witnessed some success in its short history, signing big-name stars like George Best and Pelé, but the acquisition of these high-profile players led to its downfall, where it was plagued by financial problems, following which it was disbanded in 1984 (23, 24). The successor to this league, MLS, was founded in 1996 and has grown to be financially sustainable. Over time, it has expanded, and at the time of writing, it has clubs in 18 different US states and three Canadian provinces.

In the mid-1990s, the first national women's soccer league in the United States was established. This league was still only semi-professional, and it was not until after the United States hosted the 1999 women's World Cup did a professional league come to fruition (25). Interestingly, US soccer announced this league only two days prior to the final match of the 1999 World Cup. Therefore, claims that the tournament causally increased the popularity of women's soccer, leading to this league's foundation, need to be tempered at best. However, it enabled a professional women's league to become a reality in 2001, and despite this league being short-lived, liquidating in 2003 with over $100m in cumulative debts, it was an important stepping stone to establishing a permanent professional women's league. Between 2007 and 2012, there was another failed professional league, but in 2012, the National Women's Soccer League (NWSL) was created. This league has been successful and is still running.

Soccer is now thriving in the United States, and Hopkins (26) states that the resurgence is attributable to the 1994 FIFA World Cup. It is argued that hosting this competition introduced new fans to the sport, and after following the national team, supporters embraced their local clubs, following the game regularly (27, 28). Therefore, the 1994 World Cup was a tipping point in US soccer, offering the opportunity to capitalise on the success of the tournament and reset the sport in the country. Thus, this shock had the power to influence not only the popularity of soccer in the United States, but also the spread of this popularity.

As Hopkins (26) claims, the 1994 World Cup offered the United States one last chance for acquiring greatness. If the United States got it right, the sport would take off, but if it got it wrong, it was all over for soccer in the country. The objective to stage the best World Cup ever was successful, although how this claim was measured and substantiated is contentious. But in the summer of 1994, it was declared that there was no other place to be or any other sport to watch in the United States. Following on from the World Cup, this euphoria remained, with attention diverting to the launch of MLS. Clearly, the hosting of the 1994 World Cup provided a positive shock to US soccer.

Nearly 30 years have passed since the 1994 World Cup, and the game now has far more fans who watch and play the sport than it did three decades ago. Subsequently, the United States hosted the women's tournament in 1999, and again in 2003. In 2026, the United States will co-host the men's tournament alongside Canada and Mexico. Whilst MLS has expanded and a professional women's league is flourishing, there still exists geographical inequality in terms of the general interest in the sport.

### 2.2. Soccer and political ideology

A positive correlation exists between states that vote Democrat in Presidential elections and the popularity of soccer. As CNN (10) reported, soccer is most popular in blue states and least popular in red states. There are several reasons that attempt to explain this correlation, many of which are socio-economic. Indeed, it is difficult to determine which way the causality runs, as Democratic Party supporters could have become soccer fans just as equally as soccer fans may have become more Democratic.

For example, during the 1990s, soccer in the United States was predominantly played and viewed among the Hispanic population (29), and Jensen and Limbu (30) show that Hispanics have a greater attachment to soccer than Caucasians. As de la Garza and Cortina (6) show the Hispanic population regularly vote Democrat; therefore, demography may partly explain this correlation.

In addition, women's soccer is incredibly well followed in the United States. The women's team is very successful, earning the US Soccer Federation more revenue than the men's team (31). In terms of participation during the sample period, there were approximately 348,000 registered girls playing soccer, in comparison with only 317,000 boys in 2001 (25). Contemporary data show that women's soccer remains popular in the United States, with over 400,000 female high-school players, and from the three million youth players, 48% are female (32). To put this in perspective, across all of Europe, there are only 1.27 m female players registered in total (33). With such a strong female following, soccer may gravitate to US states that are more open to female sports, or more feminist in their views, traditionally blue states.

Even after the introduction of Title IX—a US governmental policy to protect people from discrimination by sex in educational programs and activities—compliance with this law varies across the nation.^5^ After 50 years of this legislation, there are a number of US colleges that fall well short of providing enough female roster spots to conform with regulation. From the 20 least-compliant schools, 80% were found in Republican states.^6^ In addition, Lindner and Hawkins (34) outline that Democrats embrace soccer more as a sport than Republicans, who view the sport as un-American. Given that Lindner and Hawkins (34) also show that being male is a strong predictor of seeing soccer as “un-American,” it further demonstrates how soccer may have been directed towards Democratic states.

Female soccer players often face unjust prejudice and face a tough dilemma. To excel in soccer, they have to exhibit masculine traits (35). However, by exhibiting these traits or participating in a masculine sport, female athletes have their sexuality questioned (36). This can disempower female athletes and sports, potentially forcing decision-makers to place these sports into open-minded regions. As soccer is a sport perceived to be masculine (37), it may be promoted in states that strongly protect lesbian, gay, bisexual, and transgender (LGBT) rights. Given that these anti-discriminatory laws are the strongest in states that vote Democrat (38), it may be one of the reasons why soccer is most popular in blue states.

Despite the organisation and rules of soccer being created by English public schools, it is traditionally a working-class game (39). Baker (15) describes how the shortening of the working week, to a half-day Saturday, helped the working classes enjoy additional leisure time. They often watched local sides play soccer, and the sport was popular because it could be played by anyone regardless of size and strength. Therefore, its accessibility made it an inclusive game.

Traditional working-class voters hail from former Northern mill towns in England, which is where many of the oldest soccer clubs and leagues originated. Therefore, it is unsurprising that soccer in England was traditionally followed by those with political views on the left of the political spectrum. However, soccer does have a following from those who share more conservative political views. Despite their minority, there are supporter groups—self-labelled ultras—who often have nationalistic political views from certain fringe parties that follow soccer (40–42). Whilst the United States is very much a two-party system, what conjoins those on the left and right in nations with multi-party settings is often their poorer socio-economic status. Blanchflower (43) labels these individuals “the disillusioned,” or “left-behinds,” who believe the modern economic system has failed them.

Just like the wider economy, soccer has become increasingly commercialised, much to the dislike of soccer fans (44). In response, from the early 1990s, many independent supporter associations have formed to protect the interests of traditional fans (45). Therefore, soccer's modern shift appears to have moved more centrally, occupying the space close to neo-liberalism (46). In the context of the United States, this is far closer to the Democratic Party line than the Republican, indicating why we see the strong relationship between soccer's popularity and the blue states.

On the other hand, the existing literature does disprove the theory that where democrats lead, soccer follows. For example, Jewell and Molina (29) show that cities with high Hispanic populations had lower MLS attendances. Potentially this was due to the fact that MLS did not cater well for the Hispanic population (47), but it may question the idea that soccer will be successful by placing it in states with existing preconceptions in mind. In this instance, just because the Hispanic population may be passionate about soccer, positioning it in a Hispanic area will not guarantee that the sport will flourish. Thus, given that the Hispanic population tends to vote more Democratically, it potentially questions the idea that Democrats came first and then soccer.

It is claimed that soccer is a “rich white kid sport.” Allison and Barranco (48) tested whether this quote was true, finding that the hometowns of professional NWSL players had a greater white population and a higher per capita income than the national averages. Yet, Collet (11) found evidence to challenge the view that soccer fans are just “smug, liberal, elites.” Indeed, self-identified liberalism does not predict whether somebody is a soccer fan, conforming with the view of Pepple (49). Therefore, if policymakers attempted to target soccer in areas that were historically poor, liberal, and highly ethnically fractionalised, then it appears that this would have been a poor idea. This contradicts the view that Democratic states fuelled soccer's popularity.

If it is questioned that Democrats fuelled soccer's popularity, given the correlations between the two variables, it is possible that causality may run in the opposite direction. Therefore, we may witness a positive relationship because soccer may turn people to become more democratic.

### 2.3. Theoretical channels how soccer may impact the future vote

It is highly possible that liberals attend and participate in soccer matches more than Republicans, but it is equally possible that soccer's presence could instil more liberal qualities into a region. Soccer is inclusive and one of the cheapest sports to play (50), although in an organised format, equipment costs such as shin pads and boots can become an obstacle. However, grassroots football has a loose definition, and the sport can be played with just a ball in improvised spaces such as a street corner or local park (51). Thus, independent of income, athletes of different socio-economic backgrounds can come together and play.

The key theory that binds integration is social cohesion. This is defined where members of a community can build or strengthen existing relationships upon shared values, which in this framework is participating or jointly celebrating sport (52). As part of this theory, the contact hypothesis is the central mechanism in building these connections. The contact hypothesis states that under certain circumstances, contact between members of different groups may reduce prejudice, instil new beliefs, or build new relationships among them (53). To provide an example, during childhood if an individual receives exposure to peers from a different social class, culture, or background, it may influence their political identity in later years (54). Indeed, consistent with the contact hypothesis, the authors found that a percentage point increase in minorities in a white student's assigned school reduced their likelihood of registering as a Republican by 12%.

Soccer is a multicultural sport, where Mousa (19) found that soccer teams with both Muslim and Christian players had positive effects on the attitudes and behaviours of these players. Despite some fans disliking integration, for example, those who complained that the French World Cup Squad of 1998 was overly African, many see a team's multi-ethnic composition as a positive (55). Half of Germany's 2010 World Cup squad had a migration background, all who were well celebrated by the German people because of their success (56).

The globalisation of soccer has led to club teams signing players of different nationalities, religions, and races. An examination of the English club Leicester City, an outsider with 5,000-1 odds of winning the English Premier League in 2015–2016, revealed that the team was owned by a Thai businessman, managed by an Italian, had 18 different nationalities in its first team squad, and was located in one of England's most multicultural cities. As Williams and Peach (57) point out, almost a quarter of a million people turned out to welcome their local heroes upon their title triumph, in a city of 330,000. Pockets of locals, from a diverse set of cultural backgrounds, all celebrated soccer together on that day, with new friendships created and culture shared. Social identity was created and also a sense of belonging, which benefitted the city with an increase in international student admissions, alongside additional tourists, creating a more cosmopolitan metropolis.

Further demonstrating this idea, Lowe (58) shows that different Indian castes when collaborating on a common goal, through cricket—the nation's most popular sport—increased cross-caste friendships and reduced own-caste favouritism. The author further shows that adversarial contact reduced cross-caste interaction, showcasing that it was the type of contact via the contact hypothesis, in this instance sharing common goals, that improved integration.

Hosting a sporting mega event like the FIFA World Cup may further assist in increasing belonging or multiculturalism within a country. For example, at the very heart of its strategy, the 2018 Gold Coast Commonwealth Games aimed to transform its identity and acknowledge its diverse cultural heritage, shaped by Indigenous ownership, European settlement, two World Wars, and recent immigrants from around the world (59). As part of the event, Gold Coast ran a 12-day free culture festival that attracted over one million visitors, further demonstrating that sporting mega events do promote intercultural celebration (60).

Therefore, by using sporting mega events to help promote ethnic diversity, it may change the local population's perceptions of multiculturalism. Zhou and Ap (61) showed that after hosting the Beijing Olympics in 2008, local residents felt that they had a greater understanding of different people and cultures, and Grix (62) outlines how Germany used the 2006 World Cup to change its cultural image. However, cultural legacies are one of the known unknowns of hosting sporting mega events (63), and it may be naïve to propose that sporting mega events always do increase a sense of belonging and multiculturalism within a country. After the men's FIFA World Cup 2018, Russia has become increasingly isolated, and it is unlikely that multiculturalism will thrive after Qatar 2022.

Nevertheless, if soccer's presence acts as a signal that an area is more accepting of diverse backgrounds, it may encourage inward migration. If migrants are more liberal-minded, then the Democratic vote should increase, but they may also change incumbent preferences. If this occurs, then the Democratic vote should further increase as natives’ preferences change, as outlined by Dahl et al. (64) and Alesina and Tabellini (65). This would be in addition to changing preferences that arise directly through sport via the contact hypothesis, as outlined by Mousa (19) and Lowe (58).

Furthermore, with the success of the US women's national team, it has stimulated fan interest in the sport and made women's soccer incredibly popular (25, 31). Women's soccer has a strong following of male supporters and is said to be a safer and more inclusive environment in comparison with the male game (28). With an audience that is more balanced by gender in comparison with men's soccer, and also more inclusive (66), egalitarian views may be stimulated via the contact hypothesis, as shown by Dahl et al. (64).

The empirical evidence supports the idea that migration can change voting behaviour. Giuliano and Tabellini (67) find that historical immigration in the United States between 1910 and 1930 had a strong effect on political ideology, where US-born citizens located in counties with a higher historical immigrant share were increasingly likely to vote Democrat. However, there are caveats to this finding, where Halla et al. (68) show that the skill level of these migrants’ matter, and that unskilled immigrants increased the vote share of the far-right party in Austria. Similarly, Dustmann et al. (69) found that increased immigration led to centre-right and far-right parties receiving more votes, but immigration into the largest urban areas generated greater support for left-wing and pro-immigrant parties.

In terms of peer effects, Campos et al. (70) show that group dynamics do change individual preferences so that people become more alike. Specifically, on the political spectrum, individuals moved towards the centre and the results were robust to the idea that social groups are formed by individuals associating with people similar to them.

Likewise, Harmon et al. (71) find that politicians who sit near one another in the European Parliament, where seating is not necessarily shared by political ideology, tend to vote alike. This is further extended to the general population, as Finan et al. (72) show that individuals who migrate to areas where people vote more are more likely to vote in elections, and these results do not arise because of self-selection into neighbourhoods that determine turnout but arise because of peer effects.

Finally, Calderon et al. (73) find that African-American migration from 1940 to 1970 led to a steady influx of migrants to the North and West of the country, in contrast to the South with its segregationist position. The Great Migration led to a large increase in the Democratic vote share in these areas. Moreover, this increase was not purely explained by the increase in Black voters alone, further demonstrating that the preferences of the existing population may change. Thus, if peer effects matter, it is possible that individuals who have repeated interactions with a more diverse set of people and liberal voters, whether this is from attending sporting contests (19, 58) or residing in a more ethnically diverse neighbourhood, could alter their own preferences.

## 3. Materials and methods

### 3.1. Estimation strategy

To investigate the research question, we use data from 1980 to 2004 inclusive. As Presidential elections take place every 4 years, the dataset has seven time periods and 51 cross sections (US states), making the dataset a panel. The chosen estimation technique is difference-in-differences.^7^

Equation (1) shows the dependent variable (*Y*). The Democratic to Republican vote share is a function of several established covariates in (*X*), and the placement of soccer (*S*) is attributed to the 1994 World Cup.


(1) Y∼f(X,S)

Therefore, we look to examine changes in the Democratic to Republican vote share over time between US states that held World Cup games and the comparator group, US states that did not host the games. We assume that this is feasible, as the 1994 World Cup offered the United States an opportunity to reset soccer after the failings of prior professional leagues such as the NASL (23). Therefore, the selection of host cities in this tournament acts plausibly as an exogenous shock in the positioning of soccer geographically in the United States.

This is reasonable, as there existed a wide range of possible host locations for the 1994 World Cup, as prior to disbanding, the NASL had teams from approximately half of the US states during its existence from 1968 to 1984. Furthermore, the United States named 18 different cities as potential hosts in their initial 1994 World Cup application, of which only five were selected as final venues. In addition, four further venues were selected to host World Cup matches that were not initially considered venues. We also assume that these initial conditions that drive the identification strategy are independent of political decision-making. For example, the assumption that host selection was based on current or future political ideology is implausible, given the criteria often put in place exogenously by FIFA to be viable World Cup hosts.^8^

**Figure 1:**
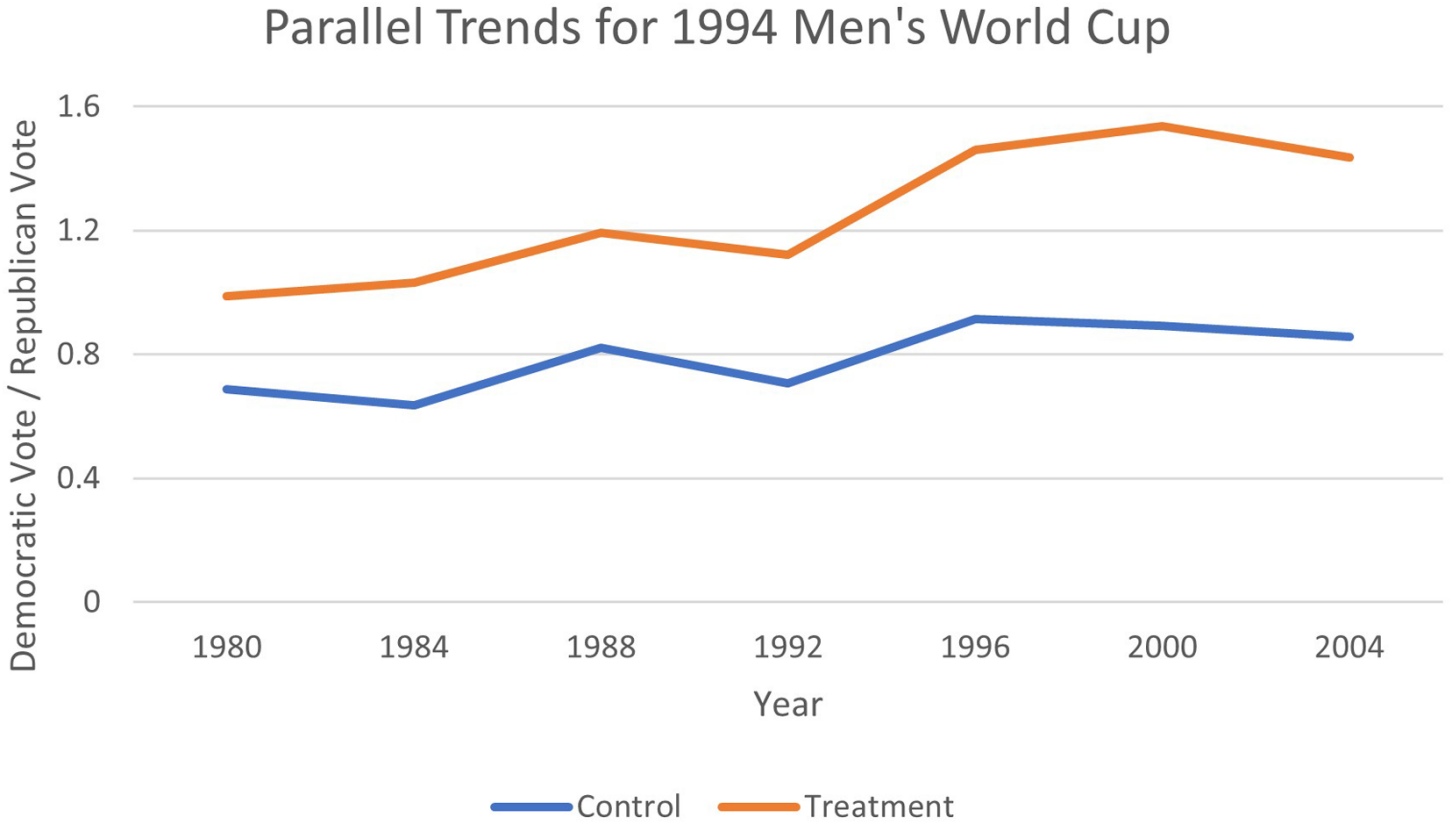
Testing parallel trends for 1994 Men's World Cup.

**Figure 2:**
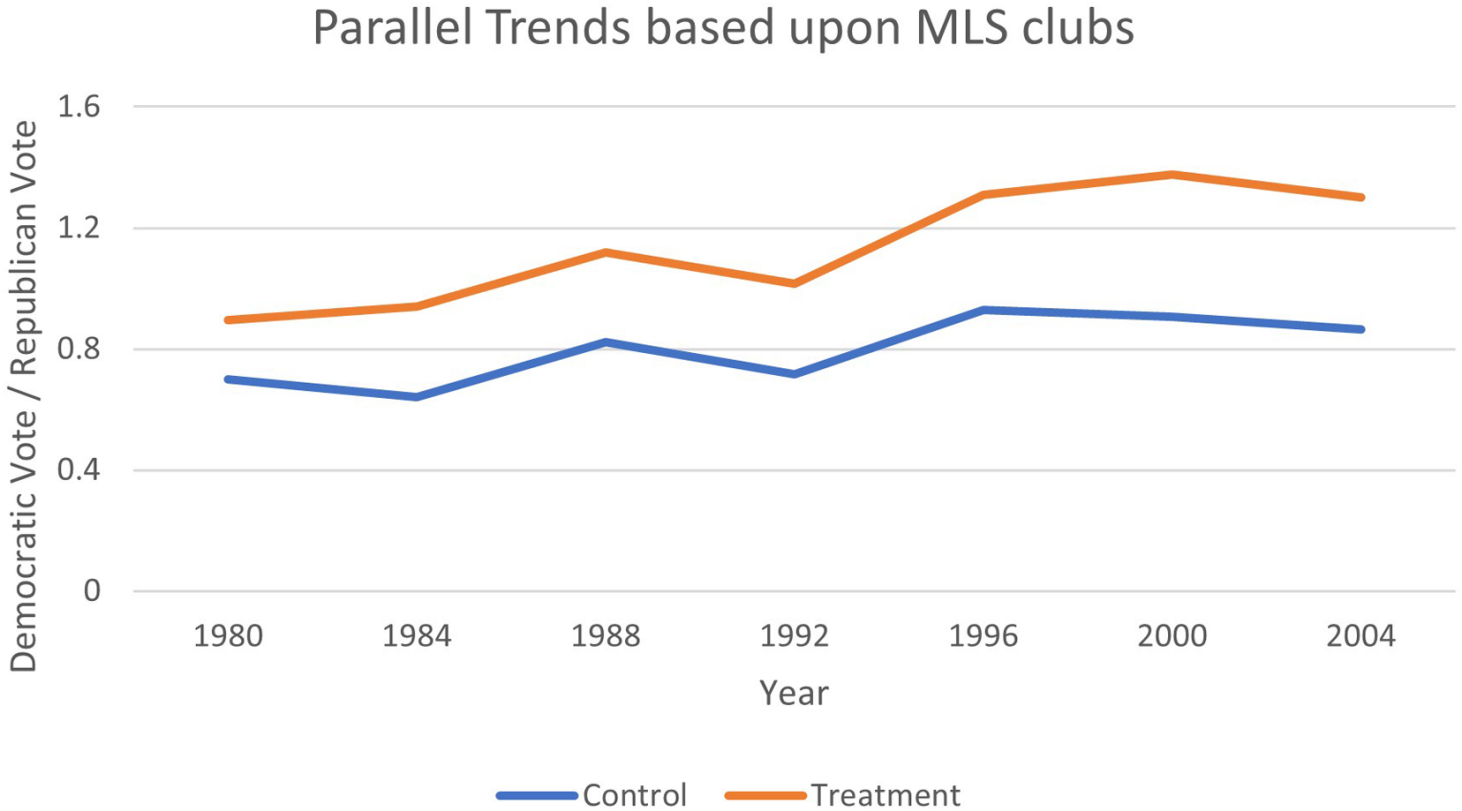
Testing parallel trends for MLS clubs.

**Figure 3:**
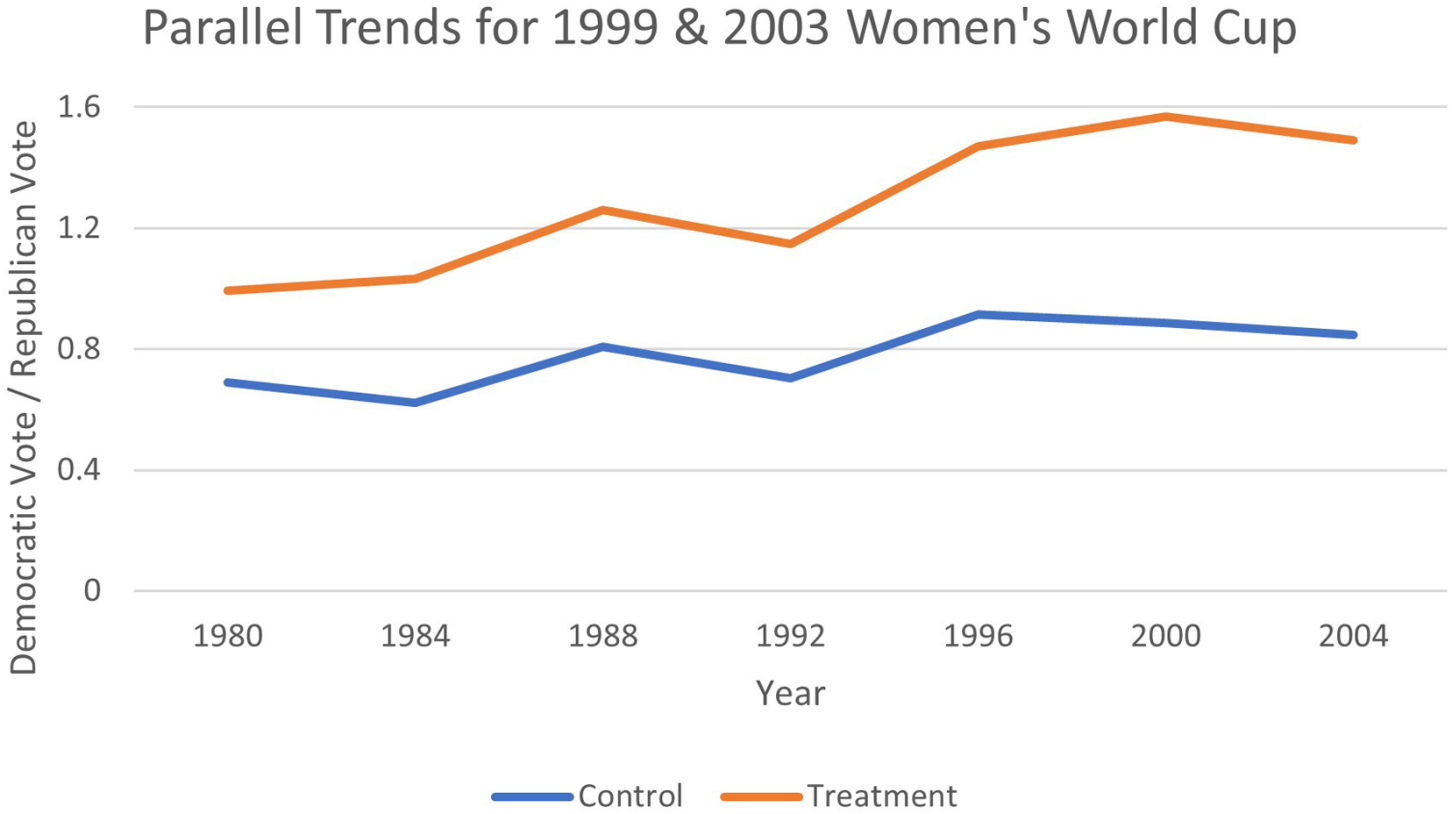
Testing parallel trends for 1999 and 2003 Women's World Cup.

**Figure 4:**
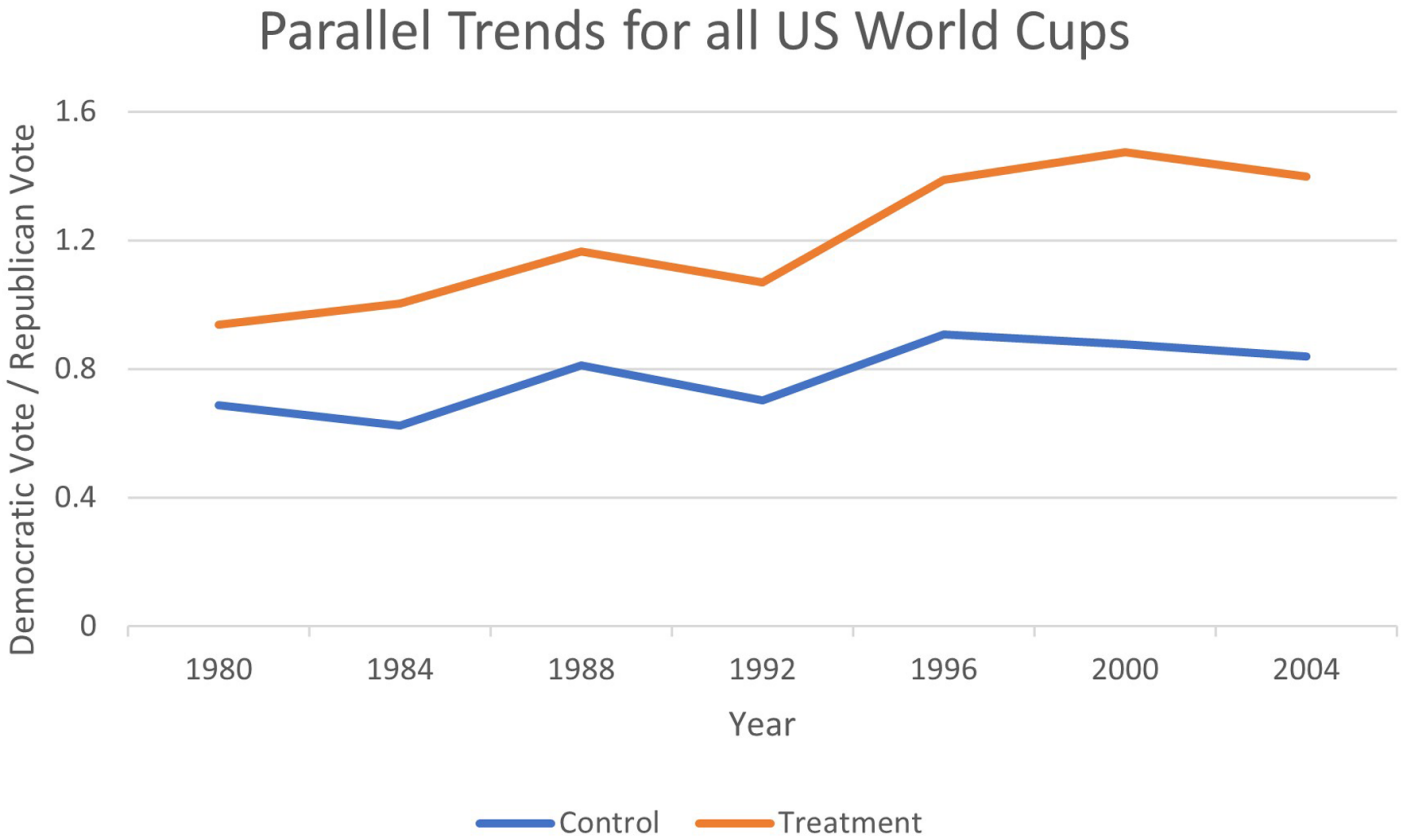
Testing parallel trends for all World Cups.

The difference-in-differences estimation strategy relies on the assumption of parallel trends. Figures 1–4 provide evidence that pre-treatment, host, and non-host states were trending at similar rates and had diverged only during the period after Soccer's introduction. Therefore, the parallel paths assumption appears to hold visually, offering evidence that the estimation technique is applicable in this context. Recently, the TWFE-DID strategy has come under scrutiny, where the negative weighting problem can reverse the signs of the treatment effect (75). To ensure that the results are not plagued by this problem, we implement the necessary tests proposed by Jakiela (76).

### 3.2. The data

The dependent variable is the total number of votes a Democratic candidate receives in a Presidential election, divided by the Republican candidate at the state level. A value equal to 1 suggests equality, where values greater than 1 indicate that the Democratic vote share exceeded the Republican share and vice versa. The data are available from the University of Santa Barbara American Presidency Project.

The 1980, 1992, 1996, and 2000 Presidential elections all had three main candidates. If the third candidate had more than 5% of the total vote share, their vote was allocated to the closest party they represented. For example, John Anderson and Ross Perot's votes were both transferred to the Republican Party given their previous affiliations.^9^ The dependent variable has a positive skew, and therefore, we winsorised six values from the upper tail of the distribution that were more than three standard deviations from the median, in order to create a normal distribution. In robustness testing, to alleviate further concerns that the dependent variable is skewed, a quantile regression evaluated at the median is used to ensure that the findings are consistent.

The variable of interest is a treatment dummy that equals 1 if a state hosted a 1994 World Cup Game and 0 otherwise.^10^ For the 1994 World Cup, the dummy equals 1 from 1996 onwards. This examines the impact of the 1994 FIFA World Cup. To examine the impact of MLS on the Democratic vote share, a dummy was created that equals 1 if a state hosted an MLS side from 1996 to 2004 from 2000 onwards.^11^ In sensitivity analysis, given that MLS was formed in 1996, the same year as a Presidential election, a treatment was also assigned for this period. Finally, to investigate the impact of the 1999 and 2003 women's World Cups, a dummy variable equals 1 for states that hosted the 1999 and 2003 women's World Cups and 0 otherwise.

The control variables are dictated by the prior literature (77, 78). They include the following: the home state of each US Democratic Presidential candidate, the home state of each US Republican Presidential candidate, the Democratic to Republican House of Representative vote share preceding a Presidential election, the share of a state's Hispanic population, the share of a state's Caucasian population, and a state's economic growth, unemployment, and inflation rates. The summary statistics, variable definitions, and sources are provided in Table 1.

**Table 1:** Summary statistics.

Variable	Mean	Standard	Minimum	Maximum	Variable source
name	value	deviation	value	value	and definition
Dem/Rep Vote Ratio	0.87	0.47	0.26	3.58	Total number of votes cast for the Democratic Party candidate divided by the total votes cast for the Republican candidate (University of Santa Barbara American Presidency Project)
Men's WC Treatment	0.08	0.26	0.00	1.00	Dummy variable equal to 1 if a state held an FIFA 1994 World Cup fixture (FIFA Website)
Major League Soccer Treatment	0.06	0.24	0.00	1.00	Dummy variable equal to 1 if a state hosted a Major League Soccer team during the sample period (MLS Website)
Women's WC Treatment	0.04	0.19	0.00	1.00	Dummy variable equal to 1 if a state held an FIFA 1999 or 2003 World Cup fixture (FIFA Website)
Dem Candidate Home State	0.02	0.14	0.00	1.00	Dummy variable equal to 1 if a state is where a Democratic Presidential candidate hails from (The United States Whitehouse Website)
)Rep Candidate Home State	0.03	0.17	0.00	1.00	Dummy variable equal to 1 if a state is where a Republican Presidential candidate hails from (The United States Whitehouse Website)
House of Representative Vote Share	0.53	0.30	0.00	1.00	The ratio of Democratic to Republican members elected in the House of Representatives by each state, preceding the previous Presidential election (history.house.gov)
Hispanic Population %	5.59	7.59	0.40	42.10	The number of Hispanic residents divided by total residents who reside within a US state in per cent (US Census Bureau)
Caucasian Population %	79.35	15.71	22.90	98.50	The number of Caucasian residents divided by total residents who reside within a US state in per cent (US Census Bureau)
State Economic Growth %	4.28	2.62	−4.40	17.14	The per cent change in state income (Bureau of Economic Analysis)
State Unemployment %	5.82	1.90	2.30	14.80	The number of individuals within a state currently out of work (US Census Bureau)
State Inflation Rate %	5.67	9.71	−3.38	35.66	The average annual percentage change in petrol prices within a State (Energy Information Administration)

Summary statistics based on 357 observations.

Following Kahane (78), dummies are included to indicate whether a state is the home state of the Democratic, and similarly, Republican candidate. For example, Arkansas was the home state for the Democratic candidate in 1992 and 1996 and received a value of 1 for these periods. This should capture the home-grown effect, with a positive coefficient expected for Democratic candidates and a negative coefficient for Republican candidates. Finally, to control for time-varying aspects of political ideology that may not be captured by the state-fixed effects, we use the Democrat percentage of votes from the House of Representatives vote in the 2 years prior to the election. These data were available from history.house.gov.

We control for ethnicity by using the percentage of a population that is (i) Hispanic and (ii) Caucasian, with the reference category as all other remaining races, including African American. These data were gathered from the US Census Bureau. As de la Garza and Cortina (6) show that Hispanics regularly vote Democrat, we anticipate this to increase the Democratic to Republican vote ratio. We also anticipate that a larger white population will decrease this ratio.

The first economic variable is the state unemployment rate and is available from the US Census Bureau. Income data at the state level are available from the Bureau of Economic Analysis and are measured as the real economic growth rate from the preceding Presidential election. To control for the state level of inflation, we examine changes in petrol prices following Kahane (78). These data are taken from the Energy Information Administration and are measured as the average annual percentage change from the preceding Presidential election.

The economic variables are required controls, but we would anticipate that they would increase the vote share for the incumbent President if the economy is performing well regardless of their political orientation. For example, low inflation, low unemployment, and high growth should provide the incumbent a higher vote share, rather than influencing the share of the Democratic vote. Only if the incumbent President was a Democrat, should we witness a positive effect. Thus, in robustness tests, we interact these economic variables with a dummy that equals 1 if a state voted for the incumbent in the previous election.

As required when using a TWFE-DID design, the specification includes state fixed effects. It is assumed that time-invariant factors, or variables that change very slowly through time within a state, determine whether it votes Democrat or Republican. Focusing on the state level is preferred to the county or city level, because it resolves the issue that cities tend to vote democratically by considering both urban and rural areas (8).^12^ In addition, there may be common shocks that influence voters' choices for all states. For example, the Watergate scandal may have driven voters in all states to vote Democrat in the 1976 election. Therefore, all specifications contain time dummies—another prerequisite of using a TWFE-DID approach.

Further controls were considered, such as income inequality and age demographics, and as Peterson et al. (79) show, voters become more conservative as they age. However, these variables were (i) relatively time-invariant and should be captured by the state fixed effects, and (ii) unavailable in panel format at the state level; therefore, we do not control for these variables. We do acknowledge that not controlling for these factors is a limitation of this study. However, one important predictor of the vote share may be the personal characteristics of the candidates. Therefore, to address this issue, we included a dummy variable for each Presidential candidate in the specification. In order to do this, we had to remove the time fixed effects to avoid perfect collinearity with the candidate dummies, as they would be common for all states in a given year. Likewise, we could not include all the candidates simultaneously; hence, we included them one at a time and ran each regression separately.

### 3.3. Additional sensitivity analysis

Several robustness tests were carried out to ensure the accuracy of the findings. In the preferred estimates, the treatment for MLS commences in 2000. This is because the league was formed in 1996, and with such a short lag between its first season and the 1996 Presidential election, there may have been insufficient time to influence the Democratic vote. Therefore, we tested if there was an immediate impact in the 1996 election from MLS, considering the 1996 period as treated.

Over the sample period, the United States hosted the Summer Olympic Games in Los Angeles (1984) and Atlanta (1996) that may potentially confound the findings. This is because the Olympics may promote multiculturalism similar to the World Cup, attracting a diverse set of visitors. Both the Los Angeles and the Atlanta Olympics also contained a soccer tournament, which may have impacted the Democratic to Republican vote ratio. Compared with the FIFA World Cup, the Olympics last 2 weeks and are concentrated in smaller regions, whereas the World Cup is played in multiple locations. Therefore, we anticipate this impact to be negligible. Nevertheless, in robustness testing, we omitted California and Georgia from the analysis.

In further robustness tests, we control for a state's previous Presidential vote, making the model dynamic by introducing a lagged dependent variable. Including this variable reduces the panel by one cross section and will add Nickell (1981) bias into the model, hence the reason why we apply this only in robustness tests. However, omitting this variable should not be concerning, given that we control for the House of Representative vote. Furthermore, as the dependent variable exhibits far more between variation than within variation, the historical vote should partially be captured by the state fixed effects.

Examining the summary statistics, it is noticeable that a number of the independent variables are skewed. In order to ensure that the results are not influenced by influential observations, we have looked to address this concern in several different ways. First, we winsorised observations more than three standard deviations from the mean and ran a number of robustness tests using these newly constructed variables. In addition, when graphically inspecting the independent variables, if there were any distributions that appeared highly skewed and a natural logarithm transform was possible, we applied this transform. The estimations were rerun using these transformed variables to see if they had any influence on the main results. As a final robustness test, we implemented a Huber regression that is robust to outliers in the dataset again checking if this had any impact on the findings.

As an extra piece of sensitivity analysis, an examination of Figures 1–4 revealed that the jump in the Democratic vote share could be attributed to the success of the first Clinton administration, when there was a surge in internet use, rise in gross domestic product (GDP) per capita, and record employment, rather than the World Cup effect. Whilst it is anticipated that these factors that would be common across all states are captured by the time fixed effects, we replace them with some select macroeconomic covariates that may better measure these confounding effects. Therefore, we include US GDP per capita, US economic growth, mobile phone subscriptions per 100 citizens, and the gross tertiary school enrolment ratio in per cent into the specification, all available from the *World Development Indicators* at the World Bank.

As a final robustness test, three falsification tests were carried out assuming that treatment started in 1996. In the first instance, treatments were randomly assigned to all US states. The second falsification test applied random treatment to seven Democratic states as outlined by the CPVI, and then the final test applied random treatment to seven Republican states judged by the CPVI.

## 4. Results

Table 2 presents the benchmark TWFE-DID results. The treatment in column 1 is positive and significant, where a state that hosted a World Cup game increased their Democratic to Republican vote ratio by 0.16 points. In column 2, the impact of MLS is investigated. In comparison with column 1, the coefficient is statistically insignificant. However, in column 3, the positive and significant effect is recovered when investigating the women's World Cup. The results show that states that hosted the games increased their Democratic vote in the Presidential elections by 0.15 points.

**Table 2:** TWFE-DID estimates on a US state's democratic vote share in US Presidential elections based on hosting a World Cup fixture or MLS team.

	(1)	(2)	(3)
Treatment	0.158**	0.069	0.152**
	(2.49)	(1.17)	(2.56)
Treatment	Men's WC	MLS	Women's WC
Controls	Yes	Yes	Yes
Fixed effects	State	State	State
Time dummies	Yes	Yes	Yes
*R*-squared	0.60	0.58	0.59
Groups	51	51	51
Observations	357	357	357

Each column represents a separate regression. The dependent variable is the ratio of each US state's popular vote between Democratic and Republican candidates, where a value of 1 equals parity or an identically shared vote. The treatment in column 1 equals 1 for all US states that hosted an FIFA Men's 1994 World Cup game. Column 2 treatment equals 1 for all US states that contain an MLS team and column 3 treatment equals 1 if a state hosted an FIFA Women's 1999 or 2003 World Cup match. Standard errors are clustered by state, where *, **, and *** represent statistical significance at the 10%, 5%, and 1% level, respectively.

A comparison of the treatment effect between the men's World Cup and the women's World Cups shows that statistically their equality is not rejected. This implies that hosting a soccer sporting event is what impacts political ideology regardless of the competition's gender.

Table 3 extends the findings to consider the joint impact of soccer events. In column 1, the impact of the men's World Cup and MLS is investigated. Column 2 examines the joint impact of the women's World Cup and MLS on the Democratic vote ratio, and column 3 investigates the impact of both men's and women's World Cups. Finally, column 4 examines the impact of all three events.^13^

**Table 3:** Joint TWFE-DID estimates on a US state's democratic vote share in US Presidential elections based on hosting a World Cup fixture or MLS team.

	(1)	(2)	(3)	(4)
Treatment	0.106*	0.059	0.136**	0.095*
Treatment variable	Men's WC and MLS	Women's WC and MLS	Women's WC and Men's WC	Women's WC, MLS, and Men's WC
Controls	Yes	Yes	Yes	Yes
Fixed effects	State	State	State	State
Time dummies	Yes	Yes	Yes	Yes
*R*-squared	0.59	0.58	0.60	0.59
Groups	51	51	51	52
Observations	357	357	357	357

Each column represents a separate regression. The dependent variable is the ratio of each US state's popular vote between Democratic and Republican candidates, where a value of 1 equals parity or an identically shared vote. The treatment in column 1 equals 1 for all US states that hosted an MLS team between 1996 and 2004. Column 2 treatment equals 1 for all US states that contain an MLS team and column 3 treatment equals 1 if a state hosted an FIFA Women's 1999 or 2003 World Cup match. Standard errors are clustered by state, where *, **, and *** represent statistical significance at the 10%, 5%, and 1% level, respectively.

In column 1, hosting the men's World Cup and/or an MLS team increases the Democratic vote ratio by 0.11 points and the variable is statistically significant at the 10% level. On the other hand, this statistically significant effect vanishes when examining states that hosted an MLS side and/or hosted the women's World Cup. In column 3 where all the World Cups are examined, the variable is positive and significant at the 5% level with a point estimate of 0.14. In column 4, the treatment variable remains statistically significant at the 10% level, but the coefficient magnitude falls to 0.10.

Where Tables 2 and 3 use a TWFE-DID estimator to investigate the research question, Table 4 extends this to explore the dynamic treatment effects, using the difference-in-differences estimator. This separates the main effect and allows us to see whether the effect is transitory or persistent, or whether it grows or shrinks over time. Allowing the effect to differ in each time period informs us whether the effect increases or dissipates, but there is a trade-off to consider. This is that the precision of the estimates is lower as we rely on inference from less data, in comparison with the traditional TWFE-DID estimator, which takes advantage of all the data in the entire post-treatment period.

**Table 4:** Dynamic difference-in-difference estimates on a US state's democratic vote share in US Presidential elections based on hosting a World Cup fixture or MLS team.

	(1)	(2)	(3)	(4)
Treatment 1996	0.141**			0.109**
	(2.41)			(2.07)
Treatment 2000	0.192**	0.097	0.152**	0.180***
	(2.19)	(1.36)	(2.21)	(2.62)
Treatment 2004	0.146**	0.041	0.130***	0.161***
	(2.41)	(0.72)	(3.11)	(3.74)
Treatment	Men's WC	MLS	Women's WC	All WC
Controls	No	Yes	No	Yes
Fixed effects	State	State	State	State
Time dummies	Yes	Yes	Yes	Yes
*R*-squared	0.60	0.58	0.59	0.60
Groups	51	51	51	51
Observations	357	357	357	357

Each column represents a separate regression. The dependent variable is the ratio of each US state's popular vote between Democratic and Republican candidates, where a value of 1 equals parity or an identically shared vote. All estimates use difference-in-differences. The treatments in column 1 equals 1 for all US states that hosted an FIFA Men's 1994 World Cup game. Column 2 treatments equals 1 for all US states that contain an MLS team and column 3 treatments equals 1 if a state hosted an FIFA Women's 1999 or 2003 World Cup match. Column 4 treatments include both FIFA men's and women's World Cups together. Standard errors are clustered by state, where *, **, and *** represent statistical significance at the 10%, 5%, and 1% level, respectively.

Column 1 shows that in all three periods, the US states that held the 1994 World Cup matches increased their Democratic vote ratio in the Presidential elections. The largest coefficient is in 2000, but you cannot reject the null hypothesis that the 1996 or 2004 treatment does not equal this value. In column 2, supporting the prior evidence, MLS has no impact on the Democratic vote ratio. When examining the impact of the women's World Cup, the largest point estimate is in 2000, but this is not statistically different from the value of the 2004 treatment period. Column 4 then investigates the impact of all the World Cups. The largest treatment effect occurs on 2000. Despite the point estimate for 1996 being the smallest and almost half the size of the 2000 treatment, the null hypothesis that the coefficients are equal is not rejected. This is also the case for the 2004 treatment.

A number of sensitivity tests were carried out to ensure the validity of the findings. First, when considering a treatment effect for MLS in 1996, the results were unchanged, with the MLS effect remaining statistically insignificant. A further concern is that the United States held the Summer Olympic Games in 1984 (California) and 1996 (Georgia) during the sample period. Given that they too may have stimulated the Democratic to Republican vote ratio, via the multicultural channel, these two states were omitted from the sample. The results remained robust.

Three placebo tests using the conventional TWFE-DID estimator were used. The first randomly assigned US states to treatment, the second randomly assigned US states to treatment that identified as pro-Democrat based on the CPVI, and finally, the same procedure was conducted for pro-Republican states. In all the placebo tests, the treatment term was statistically insignificant.^14^

A threat to identification is that US states that were Democratic (Republican) prior to the 1994 World Cup may have just become more Democratic (Republican) over time. To overcome this risk, a lagged dependent variable was entered into the specification. Whereas the lagged dependent variable was statistically significant, the treatment effect remained statistically significant.^15^ We also tested this effect by introducing a dummy equal to 1 if a state swung from the previous election. This method avoided Nickell bias, as the specification had no lagged dependent variable, yet controlled for effects in the previous period. The treatment effect remained positive and statistically significant.

As a further robustness check, to better condition for the impact of the economic variables, we interacted them with a dummy equal to 1 if a state voted for the current incumbent in the previous election. This is because a high (low) growth (inflation/unemployment) rate would be beneficial to the incumbent President regardless of their political affiliation. The results remained similar to the findings reported in Tables 2–4.

The next piece of sensitivity analysis tested whether the heterogenous treatment timings were susceptible to a negative weighting problem. As many of the venues selected for the women's World Cup in 1999 and 2003 were similar to those for the 1994 World Cup, this should not be a serious concern. Nevertheless, using the Jakiela (76) procedure of examining the weights associated with the treatment group, it was found that there was no evidence of a negative weighting problem.

The following robustness tests altered the regression specification to test if the results were susceptible to outliers given the skewed nature of the dataset, if they are confounded by the success of the first Clinton administration, and controlling for the personal characteristics of the Presidential candidates. Tables 5 and 6 showcase the main findings, replicating the results from Table 2.

**Table 5:** TWFE-DID robustness tests examining the impact on a US state's democratic vote share in US Presidential elections based on hosting a World Cup fixture or MLS team.

	(1)	(2)	(3)
Panel A: Winsorising independent variables three standard deviations from mean
Treatment	0.136*	0.047	0.132**
	(1.88)	(0.76)	(2.15)
Panel B: Applying a natural logarithm transform to the unemployment rate and Hispanic population
Treatment	0.126*	0.046	0.136**
	(1.83)	(0.77)	(2.21)
Panel C: Estimation using a Huber regression
Treatment	0.163***	0.085**	0.165***
	(4.44)	(2.15)	(3.84)
Panel D: Estimation using a Quantile regression (at median)
Treatment	0.148***	0.080	0.111*
	(2.68)	(1.49)	(1.83)
Panel E: Replacing time-fixed effects with US Macro Control Variables
Treatment	0.238***	0.054	0.134**
	(3.16)	(0.86)	(2.03)
Treatment	Men's WC	MLS	Women's WC
Controls	Yes	Yes	Yes
Groups	51	51	51
Observations	357	357	357

Each column represents a separate regression within each panel. The dependent variable is the ratio of each US state's popular vote between Democratic and Republican candidates, where a value of 1 equals parity or an identically shared vote. The treatment in column 1 equals 1 for all US states that hosted an FIFA Men's 1994 World Cup game. Column 2 treatment equals 1 for all US states that contain an MLS team and column 3 treatment equals 1 if a state hosted an FIFA Women's 1999 or 2003 World Cup match. Standard errors are clustered by state, where *, **, and *** represent statistical significance at the 10%, 5%, and 1% level, respectively.

**Table 6:** Further TWFE-DID robustness tests examining the impact on a US state's democratic vote share in US Presidential elections based on hosting a World Cup fixture or MLS team.

	(1)	(2)	(3)	(4)	(5)	(6)	(7)	(8)	(9)	(10)
Panel A: Treatment variable if a state hosted a 1994 FIFA World Cup match
Treatment	0.220***	0.236***	0.231***	0.238***	0.228***	0.216***	0.157**	0.208***	0.222***	0.225***
	(3.13)	(3.34)	(3.34)	(3.50)	(3.16)	(3.07)	(2.28)	(3.12)	(3.11)	(3.26)
Panel B: Treatment variable if a state hosts an MLS team during the sample period
Treatment	0.039	0.053	0.056	0.029	0.035	0.063	0.077	0.064	0.040	0.043
	(0.72)	(0.92)	(1.01)	(0.51)	(0.63)	(1.08)	(1.38)	(1.10)	(0.70)	(0.76)
Panel C: Treatment variable if a state hosted a 1999 or 2003 FIFA World Cup match
Treatment	0.127**	0.136**	0.139**	0.123**	0.125*	0.146**	0.155**	0.154**	0.132**	0.129**
	(2.11)	(2.17)	(2.26)	(2.02)	(1.99)	(2.33)	(2.59)	(2.43)	(2.08)	(2.15)
President	Carter	Reagan	Mondale	Dukakis	Bush Sr	Clinton	Dole	Bush Jr	Gore	Kerry
Controls	Yes	Yes	Yes	Yes	Yes	Yes	Yes	Yes	Yes	Yes
Groups	51	51	51	51	51	51	51	51	51	51
Observations	357	357	357	357	357	357	357	357	357	357

Each column represents a separate regression where the time dummies are replaced using a dummy variable for a specific Presidential candidate. The dependent variable is the ratio of each US state's popular vote between Democratic and Republican candidates, where a value of 1 equals parity or an identically shared vote. The treatment in Panel A equals 1 for all US states that hosted an FIFA Men's 1994 World Cup game. The Panel B treatment equals 1 for all US states that contain an MLS team, and in Panel C, the treatment equals 1 if a state hosted an FIFA Women's 1999 or 2003 World Cup match. Standard errors are clustered by state, where *, **, and *** represent statistical significance at the 10%, 5%, and 1% level, respectively.

Overall, Table 5 shows that the regression coefficient on the treatment variables does not differ dramatically from that in Table 2. The top panel shows the results when the variables are winsorised, transforming observations more than three standard deviations away from the mean. The next panel replaces the unemployment and Hispanic population variable with its natural logarithm. The third panel estimates the results using a Huber regression. The fourth panel then presents the findings using a quantile regression to overcome any remaining concerns about potential skewness in the dependent variable. The bottom panel then removes the time fixed effects, replacing them with certain macroeconomic variables, and shows that the findings are not sensitive to this alteration in the specification.

In Table 6, the treatment variable increases in magnitude for the men's World Cup but remains relatively similar for the MLS and women's World Cup treatment variables. Therefore, it provides further confidence in the main findings. Unreported, an examination of the impact of specific Presidents on the Democratic vote ratio revealed that George Bush Jr increased the Republican vote by 0.14 points, whereas Michael Dukakis increased the Democratic vote share the most, closely followed by Bill Clinton.

**Figure 5:**
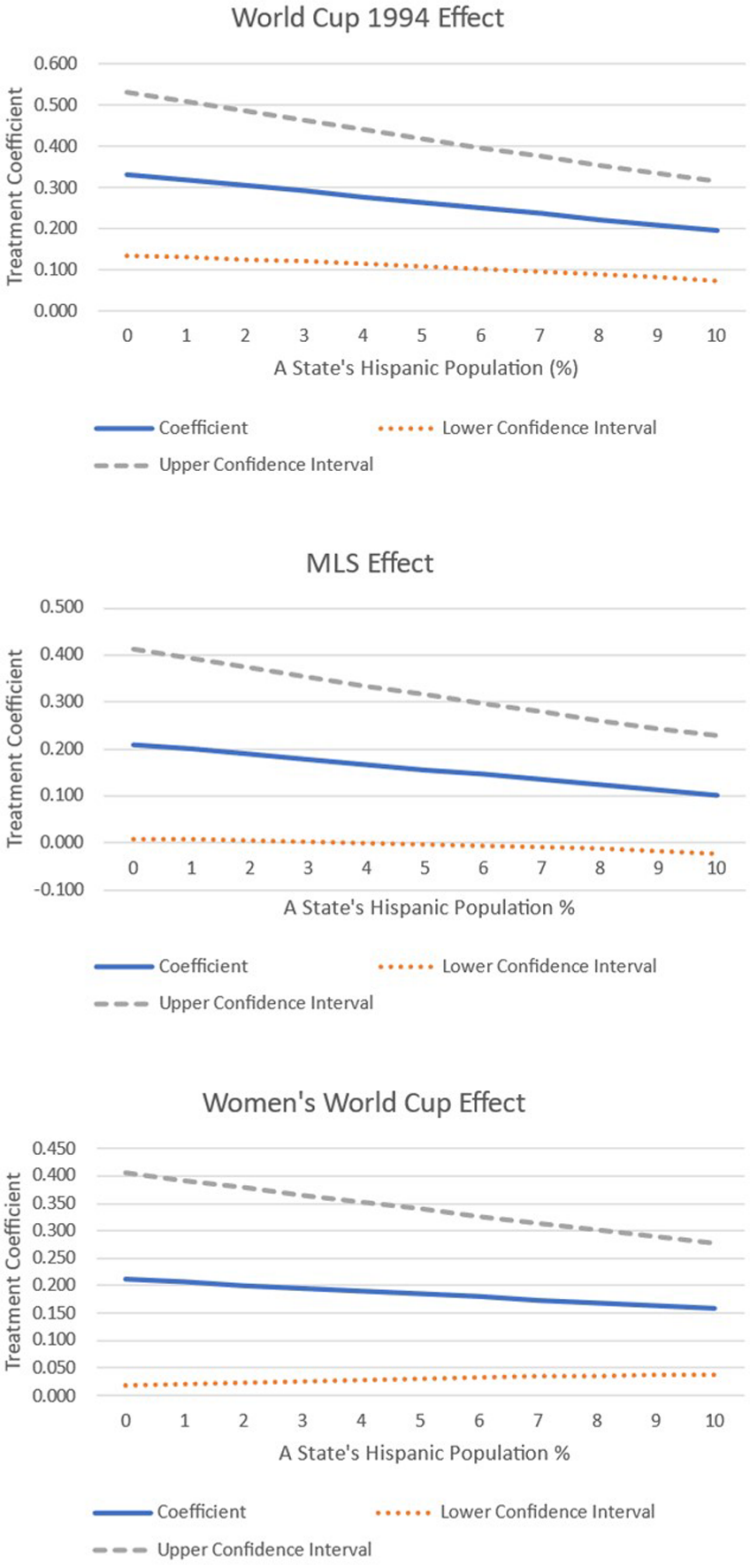
Interacting treatment effects with the size of a state's Hispanic population.

**Figure 6:**
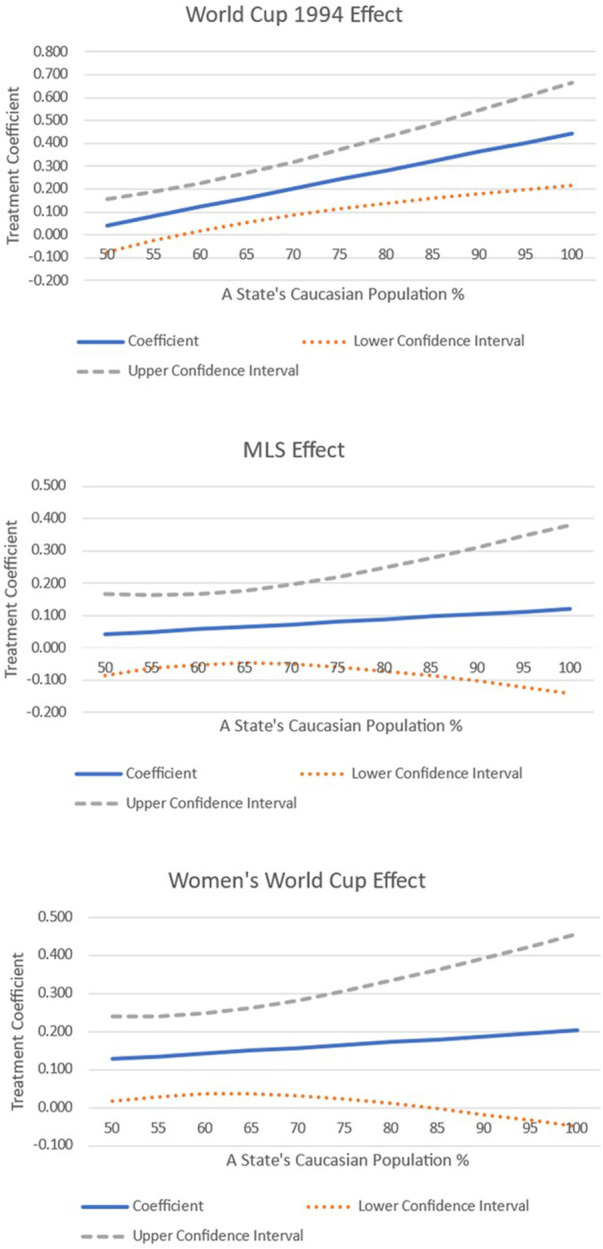
Interacting treatment effects with the size of a state's Caucasian population.

**Figure 7:**
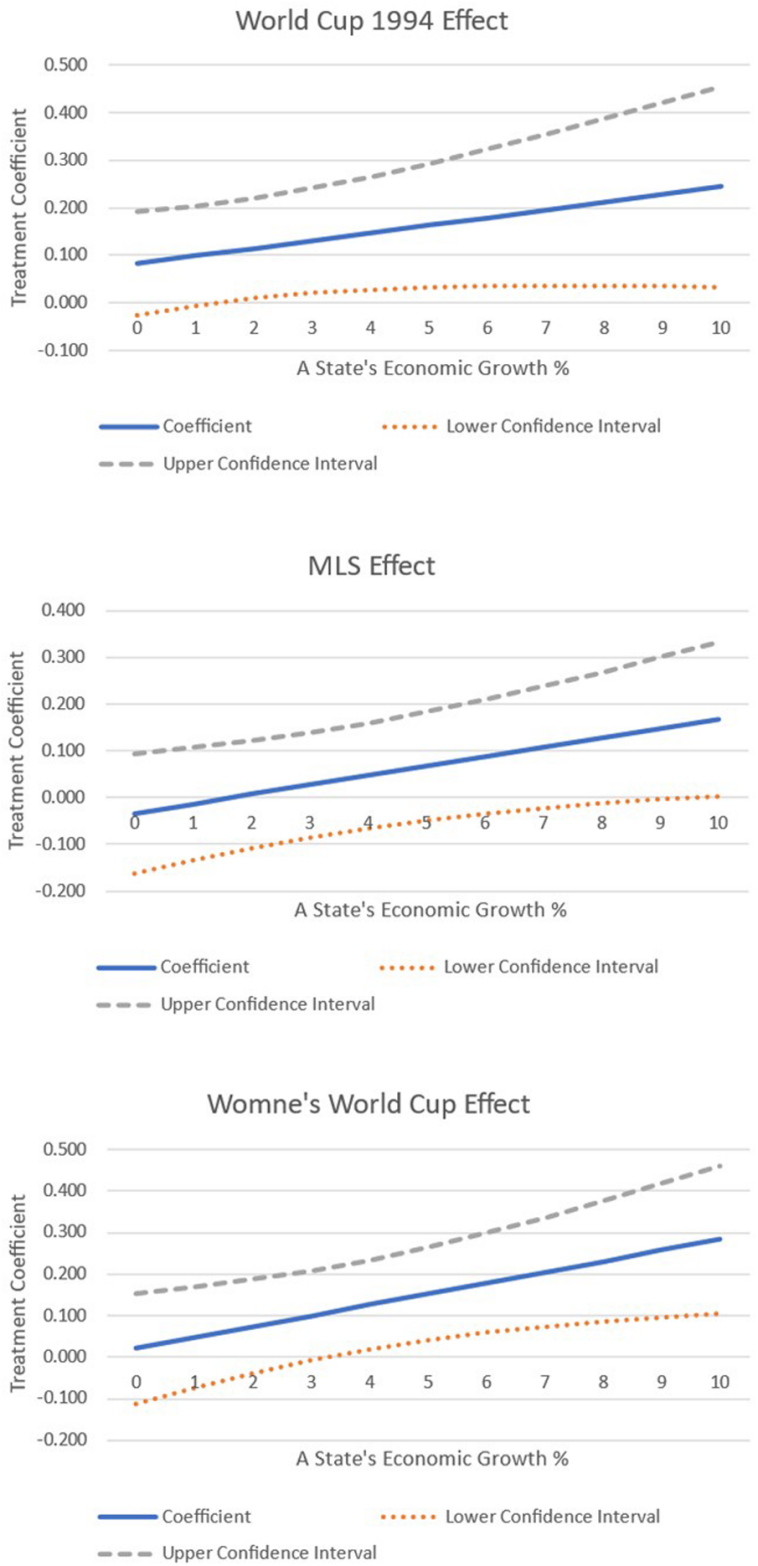
Interacting treatment effects with a state's economic growth rate.

As a final piece of analysis, we interacted the treatment variable with some of the covariates. We focused on testing whether the treatment effect varied, given the differences in ethnicity within a state, and whether it changes depending on a state's income growth. The results are shown graphically in Figures 5–7.

Figure 5 shows how the treatment varies given a state's Hispanic population. Along the *X*-axis we model this across the sample distribution ranging between 0% and 10% of the total state population. An examination of the 1994 World Cup effect reveals that the treatment is statistically significant across the entire distribution but falls in magnitude as the Hispanic share of the state population increases. Likewise, when examining the impact of hosting an MLS franchise, it is found that only if the Hispanic share of the total population equals 4% or less is there a positive treatment effect, outlined by the lower confidence interval crossing 0 beyond this point. Finally, when examining the impact of the women's World Cup, a similar pattern emerges, as when the Hispanic share of the population effect increases within a state, the magnitude of the treatment falls.

Figure 6 focuses on how the treatment effect changes when interacting it with our second ethnicity variable, the size of the Caucasian population within a state. The results show that as the percentage of Caucasians increases in a treated state, the impact of soccer on the Democratic vote share also increases. The threshold for when it becomes significant for the 1994 World Cup is when the proportion of the Caucasian population in a state is 60% or greater. Interestingly, there is no impact throughout the distribution when the treatment focuses on cities that host MLS teams. Finally, when examining the treatment effect of hosting the women's World Cup, it is found that whilst the coefficient increases in magnitude when there is a greater Caucasian population in a treated state, the effect does become statistically insignificant when the Caucasian population goes beyond 80% of the total population.

Finally, Figure 7 shows that the higher the income growth in treated states, the greater the impact of hosting on the Democratic vote share. When a treated state's income growth is 2% or greater, the Democratic vote share increases as a result of hosting a 1994 World Cup match. When examining the impact of MLS, it is found that only when income growth is 10% at the very top end of the distribution is there any impact on the Democratic vote share in treated states. Finally, when examining the impact of hosting a women's World Cup match, it is found that only when treated states have their income growing at 4% or more does the Democratic vote share increase.

## 5. Discussion

The 1994 FIFA World Cup provided the United States an opportunity to capitalise on an international soccer tournament. It had the power to influence not only the popularity of soccer in the United States but also the spread of this popularity. Specifically, it provided host cities an opportunity to act as focal areas for soccer to grow. Soccer's multicultural aspects can drive greater ethnic tolerance and liberal thinking, which could be spread by the mechanisms of migration and changing preferences. Social cohesion theory and the contact hypothesis are the main mechanisms that would drive these changes (52, 53). Sport is a setting where these ideas are shown to have a large impact, as shown by both Mousa (19) and Lowe (58) that collaborating on a common goal does lead to greater integration. Thus, it is possible that soccer could make you more Democratic.

The results show that those US states that held men's World Cup fixtures increased their Democratic vote share in subsequent Presidential elections, lending support to the hypotheses mentioned previously. A condition of hosting the 1994 World Cup was that the United States had to create a professional men's league. However, US states that hosted MLS teams from 1996 to 2004 did not increase their Democratic vote share over this time period. This is interesting because it suggests that international effects dominate domestic effects. Potential reasons for this include that international soccer events will result in more cultures coming together, or alternatively, being an international host may signal openness to the world (80). There is empirical evidence supporting this view, as after hosting the Beijing Olympics in 2008, Zhou and Ap (61) found that local residents had a greater understanding of different people and cultures. Nevertheless, this may not be the case for every sporting mega event, as it is unlikely that cultural diversity will flourish post-Qatar 2022.

A further finding shows that the increase in the Democratic vote share from hosting the women's World Cup is similar to that from hosting the men's World Cup. There is a great deal of variation between women's rights across US states, most noticeable in recent times with the overturning of Roe v Wade (81); therefore, it is interesting that these effects are equal. For example, states that hosted the women's tournament may have been perceived to be exceptionally progressive, attracting more liberal voters via migration, who move closer to individuals similar to them via the concept of homophily (16, 17). Alternatively, hosting women's sporting contests that are found to be more inclusive (28) may facilitate an increase in liberal attitudes, as found by Dahl et al. (64), which may manifest in the ballot booth. However, the evidence does not support this view given the similar values of the coefficients.

US voting behaviour has also been found to be impacted by certain socio-economic characteristics (4–7). Interacting the ethnicity and economic variables with the treatment effect led to some interesting findings. First, when the Hispanic population is low in a treated state, the effect is the largest and falls in magnitude when the Hispanic population in a treated state reaches a rate of 10%.

This is interesting, as Jensen and Limbu (30) suggest that Hispanics have a greater attachment to soccer than Caucasians, and during the sample period, soccer in the United States was predominantly viewed among this community (29). Given the findings of de la Garza and Cortina (6) who show that the Hispanic population regularly vote Democrat, this result may be unsurprising. This is because if a state already has a large Hispanic population, and is already voting Democrat, then at the margin, any increases in the Democratic vote should be smaller.

The second ethnicity interaction shows that when the treatment is interacted with the Caucasian population, the findings differ between whether the state hosted a men's or women's World Cup. Specifically, if the Caucasian population is greater than 60% of the total population, the Democratic vote share increases and grows for treated states. On the other hand, the effect vanishes for treated states that hosted the women's World Cup when the Caucasian population becomes greater than 80% of the total population.

Linking this with Allison and Pope (28) who suggest that women's soccer often has a more diverse audience, this may explain the above finding. With less initial cultural fractionalisation within a state, soccer matches would also be less diverse. This would mean that individuals will make contact with those similar to them and not have the opportunity to experience different cultures, potentially blocking the contact hypothesis (19, 58). Nevertheless, as Allison and Barranco (48) find, professional NWSL players typically come from hometowns that have a greater white population, contrasting the evidence from this paper. Therefore, with two opposing forces at play, to identify precisely what is going on is something worth exploring in future research with household data.

Similarly, Allison and Barranco (48) show that the hometowns of professional NWSL players have a higher per capita income than the national average. Therefore, if soccer does lead to an increase in the Democratic vote share, it is plausible to assume that it may have a greater impact on treated states that are wealthier or are growing in economic prosperity. The results seem to confirm this finding, where once income growth surpasses a certain threshold, only then does soccer impact the Democratic vote share. Furthermore, this impact increases with income growth, and the magnitude of the coefficients between hosting a men's or women's World Cup remain very similar. Given the popularity of women's soccer in the United States (31), this finding conforms to our expectations.

It is well established that there is a strong correlation between those who follow soccer and those who vote Democrat. Yet, which direction does this relationship run? Is soccer drawn to regions that are more liberal, or does soccer entice areas to become more liberal? This research refutes the claim of unidirectional causality that Democratic states came first and adopted soccer. This is because the results strongly support that soccer increased the ratio of the Democratic to Republican vote after its plausibly exogenous positioning. However, this paper does not claim that causality only runs from soccer to politics. This relationship could be bi-casual and this study offers a platform to study which direction the causality does run.

This research does have its limitations. Whilst the shock of 1994 is plausibly exogenous, there is an element of non-randomness. For example, there were certain locations in which conditions were more favourable to host the 1994 World Cup, dictated by their existing infrastructure with major airports and abundant hotel space. These locations may already have been pre-existing tourist hubs with a cosmopolitan feel and already embraced multiculturalism, one of the theoretical mechanisms central to this paper.

Linked to this limitation, it would be of further interest to explore the mechanisms that drive the changes in the Democratic vote that arise through soccer. However, without an extensive household dataset, it is challenging to test these channels directly. Whilst we have managed to test differences on the basis of ethnicity and income growth, we have had to rely on bulky aggregate indicators at the state level. Being able to examine this relationship by social class, income, and even gender at the micro level may provide further interesting insights within this topic.

A further limitation is that other shocks over the sample period may have influenced the Democratic to Republican vote ratio. For example, political scandals involving Senators or Representatives within a US state may influence how that state would vote in the next Presidential election.^16^ This issue may never be controlled completely, but conditioning for each state's House of Representatives vote prior to the next Presidential election should limit this concern by capturing any time-varying shifts in political ideology.

Finally, the generalisation of these findings over time may be questionable. It is worth noting that participation and viewership of soccer is far greater today than it was in 1994. Therefore, the effects in 2026 may be very different from what the results show in the past. Furthermore, the United States has witnessed an increase in political polarisation during recent years, and the political climate is different from what it was during the sample period (20, 21). How this may impact the future vote is debatable. But if the contact hypothesis has been pivotal in the decision-making of median voters who have already changed their preferences, or will impact those median voters who are planning to realign themselves with a new political party, then the role of soccer could still shape political ideology.

In conclusion, this research attempts to explain the uneven spread of soccer in the United States. Claims that the nation is split politically are well substantiated, and geography plays it part, where certain regions of the country strongly vote Democratic and others Republican. This political polarisation has been suggested to explain the uneven spread of soccer in the United States, where it has flourished in Democratic strongholds.

This paper refutes the uni-directional causality arguments that politics has driven soccer's placement in the country. Indeed, it shows that hosting international soccer events may even influence political ideology. Whilst this paper does not attempt to forecast future Presidential results, if soccer does make you more left wing, will the United States, which is co-hosting the 2026 FIFA men's World Cup, find certain host areas become more liberal? If so, pay attention to the swing states Georgia and Florida, both of which will be hosting World Cup matches.

## Data Availability

The raw data supporting the conclusions of this article will be made available by the authors without undue reservation.

## Footnotes

^1^The popularity of soccer in this context could be one of any number of things such as participation, viewership both in person or on television, or just generic interest in the sport. Most of these facets typically will be highly correlated with one another, and when loosely used in this manuscript, reflect an appetite for the sport.

^2^MLS currently has 28 clubs at the time of writing with a 29th franchise to be admitted in 2023.

^3^The United States acted as emergency hosts in 2003 following the SARS outbreak in China.

^4^In total, 14 US states have all hosted the men’s and women’s World Cup and had an MLS side from 1996 to 2004. From these, only five uniquely hosted one of the three events.

^5^In full, Title IX states: “No person in the United States shall, on the basis of sex, be excluded from participation in, be denied the benefits of, or be subjected to discrimination under any education program or activity receiving Federal financial assistance.”

^6^Data available from USA Today available at: https://eu.usatoday.com/in-depth/news/investigations/2022/06/03/title-ix-failures-50-years-colleges-women-lack-representation/9664260002/.

^7^We direct the reader to (74) Chapter 5 for those who wish to gain a more comprehensive understanding of the estimation technique.

^8^Whilst politicians may try to position host cities in areas that require economic development, academic research often finds limited to no economic benefits of hosting a sporting mega event. Therefore, it is unlikely voters would have reacted to this, and if they had, it would only benefit the incumbent party at the time, irrespective of their ideology.

^9^In robustness testing, we assumed that the vote was split equally to each candidate as in Fair (77).

^10^We assign both standard treatment effects for the TWFE-DID estimator and dynamic treatments when implementing a dynamic difference-in-differences design.

^11^As Presidential elections are held every 4 years, the data are spaced every 4 years from 1980 to 2004 inclusive. Therefore, there are three treatment periods for the men’s World Cup from a total of seven periods and two treatment periods for MLS hosts and the women’s World Cup.

^12^For example, the state of South Carolina, over the past 40 years and 11 elections, has provided its electoral college votes to the Republican Party, yet the city of Columbia leans in favour of the Democratic Party and has preferred the Democratic candidate in the last six elections.

^13^When examining multiple soccer events together, the treatment varies across both units and time. Examining dynamic treatment effects may abate the timing issue, but whether this invalidates the TWFE-DID estimator may be tested using a procedure outlined by de Chaisemartin and D'Haultfœuille (76).

^14^When examining the dynamic difference-in-difference effects under a placebo in the randomly allocated pro-Democrat group, the 2000 and 2004 treatments were statistically significant at the 10% level. This was further investigated in robustness tests examining persistence.

^15^The introduction of a lagged dependent variable with fixed effects may cause Nickell bias, and that is why these regressions are only robustness tests. In addition, we hope that this evidence shows that the statistical significance in the dynamic difference-in-difference placebo test for pro-Democrat states is perhaps just an anomaly.

^16^For example, Dan Rostenkowski’s corruption charges in the House Post Office Scandal may have influenced voters in Illinois to shift from Democrat to Republican in the 1996 election following his conviction in 1994.

## References

[1] Hirano S Snyder JM Jr. The decline of third-party voting in the United States. J Polit. (2007) 69(1):1–16. 10.1111/j.1468-2508.2007.00490.x

[2] Garand J. Income inequality, party polarisation and roll-call voting in the U.S. Senate. J Polit. (2010) 72(4):1–16. 10.1017/S0022381610000563

[3] Grossmann M Hopkins DA. Ideological republicans and group interest democrats: the asymmetry of American party politics. Perspect Polit. (2015) 13(1):119–39. 10.1017/S1537592714003168

[4] Axelrod R. Where the votes come from: an analysis of electoral coalitions, 1952–1968. Am Pol Sci Rev. (1972) 66(1):11–20. 10.2307/1959275

[5] Jackson J. Issues, party choices and presidential votes. Am J Pol Sci. (1975) 19(2):161–85. 10.2307/2110431

[6] de la Garza R Cortina J. Are Latinos republicans but just don’t know it? The latino vote in the 2000 and 2004 presidential elections. Am Polit Res. (2007) 35(2):202–23. 10.1177/1532673X06294885

[7] Curtis L Hoffman M Califf R Hammill B. Life expectancy and voting patterns in the 2020 U.S. Presidential election. SSM Popul Health. (2021) 15:100840. 10.1016/j.ssmph.2021.100840 34169139 PMC8209240

[8] Frank T. What's the matter with Kansas: How conservatives won the heart of America. New York: Metropolitan Books (2004).

[9] US Youth Soccer (2012). Available at: www.usyouthsoccer.org/media_kit/keystatistics/.

[10] CNN. Willingness to embrace soccer’ reflects a shift in the US (2014). Available at: https://cnnpressroom.blogs.cnn.com/2014/07/06/peter-beinart-willingness-to-embrace-soccer-reflects-a-shift-in-the-u-s/.

[11] Collet C. Soccer, politics and the American public: still “exceptional”? Soccer Soc. (2017) 18:348–67. 10.1080/14660970.2016.1166766

[12] Russell D. Associating with football: social identity in England 1863–1998. In: Armstrong G, Giulianotti R, editors. Football cultures and identities. London: Palgrave Macmillan (1999.

[13] Woods J. Red sport, blue sport: political ideology and the popularity of sports in the United States. Int J Sport Policy Politics. (2022) 14(3):489–505. 10.1080/19406940.2022.2074516

[14] EFL. English Football League (2022). Available at: https://www.efl.com/-more/all-about-the-efl/history/#:∼:text=The%20twelve%20founder%20members%20of,Bromwich%20Albion%20and%20Wolverhampton%20Wanderers.

[15] Baker W. The making of a working-class football culture in Victorian England. J Soc Hist. (1979) 13(2):241–51. 10.1353/jsh/13.2.241

[16] Currarini S Jackson MO Pin P. An economic model of friendship: homophily, minorities, and segregation. Econometrica. (2009) 77(4):1003–45. 10.3982/ECTA7528

[17] Campigotto N Rapallini C Rustichini A. School friendship networks, homophily and multiculturalism: evidence from European countries. J Popul Econ. (2022) 35(4):1687–722. 10.1007/s00148-020-00819-w

[18] Gilligan MJ Pasquale BJ Samii C. Civil war and social cohesion: lab in the field evidence from Nepal. Am J Pol Sci. (2014) 58(3):604–19. 10.1111/ajps.12067

[19] Mousa S. Building social cohesion between Christians and Muslims through soccer in post-ISIS Iraq. Science. (2020) 369(6505):866–70. 10.1126/science.abb3153 32792403

[20] Gelman A Goel S Rivers D Rothschild D. The mythical swing voter. Quart J Polit Sci. (2016) 11(1):103–30. 10.1561/100.00015031

[21] Duca JV Saving JL. Income inequality, media fragmentation, and increased political polarization. Contemp Econ Policy. (2017) 35(2):392–413. 10.1111/coep.12191

[22] Crawford S. A history of soccer in Louisiana: 1858–2013. United States: LAprepSoccer (2013).

[23] Watanabe N. The economics of major league soccer from the NASL to MLS: a brief history of north American professional soccer. In: Downward P, Frick B, Humphreys BR, editors. The SAGE handbook of sports economics. SAGE Publications Ltd (2019). p. 331.

[24] Rewilak J. The designated player policy rule and attendance demand in major league soccer. J Sports Econom. (2022):15270025221134234. 10.1177/15270025221134234. [epub ahead of print]

[25] Markovits A Hellermen S. Women’s soccer in the United States: yet another American exceptionalism. Soccer Soc. (2003) 4:14–29. 10.1080/14660970512331390805

[26] Hopkins G. Star spangled soccer. The selling, marketing and management of soccer in the USA. New York: Palgrave-Macmillan (2010).

[27] Gerke M. For club and country? The impact of the international game on US soccer supporters from the 1994 World Cup to the present. Soccer Soc. (2019) 20(5):770–9. 10.1080/14660970.2019.1616269

[28] Allison R Pope S. Becoming fans: socialization and motivations of fans of the England and US women's national football teams. Sociol Sport J. (2021) 39(3):287–97. 10.1123/ssj.2021-0036

[29] Jewell T Molina D. An evaluation of the relationship between Hispanics and major league soccer. J Sports Econom. (2005) 6(2):160–77. 10.1177/1527002504263400

[30] Jensen RW Limbu YB. Soccer fans’ motivations, attitudes, and behavioral intentions across ethnicity and gender lines: are Hispanics in the United States more passionate about soccer than Caucasians? Public Policy Adm. (2016) 4(1):43–57. 10.15640/ppar.v4n1a5

[31] Culvin A Bowes A Carrick S Pope S. The price of success: equal pay and the US women’s national soccer team. Soccer Soc. (2022) 23(8):920–31. 10.1080/14660970.2021.197728031619942

[32] Statista (2021). Available at: https://www.statista.com/statistics/267963/participation-in-us-high-school-soccer/.

[33] UEFA (2019). Available at: https://www.uefa.com/insideuefa/football-development/womens-football/.

[34] Lindner AM Hawkins DN. Globalization, culture wars, and attitudes toward soccer in America: an empirical assessment of how soccer explains the world. Sociol Q. (2012) 53(1):68–91. 10.1111/j.1533-8525.2011.01226.x

[35] Allison R. Kicking center: Gender and the selling of women’s professional soccer. New Brunswick, NJ: Rutgers University Press (2018).

[36] Blinde EM Greendorfer SL Shanker RJ. Differential media coverage of men's and women's intercollegiate basketball: Reflection of gender ideology. J. Sport Soc. Issues. (1991) 15(2):98–114. 10.1177/01937235910150020

[37] Scheadler T Wagstaff A. Exposure to women’s sports: changing attitudes toward female athletes. Sport J. (2018) 19:1–17. https://thesportjournal.org/article/exposure-to-womens-sports-changing-attitudes-toward-female-athletes/

[38] Hasenbush A Flores A Kastanis A Sears B Gates G. The LGBT divide: A data portrait of LGBT people in the midwestern, mountain & southern states. Los Angeles: The Williams Institute, UCLA School of Law (2014).

[39] Taylor M. The association game: A history of British football. London: Routledge (2008).

[40] Armstrong G Testa A. Football, fascism and fandom: The ultras of Italian football. London: A and C Black Publishers Ltd (2010).

[41] Djordjević I Pekić R. Is there space for the left? Football fans and political positioning in Serbia. Soccer Soc. (2018) 19(3):355–72. 10.1080/14660970.2017.1333678

[42] Woźniak W Kossakowski R Nosal P. A squad with no left wingers: the roots and structure of right-wing and nationalist attitudes among Polish football fans. Probl Post-Communism. (2020) 67(6):511–24. 10.1080/10758216.2019.1673177

[43] Blanchflower D. Not working: Where have all the good jobs gone? NJ, Princeton: Princeton University Press (2019).

[44] Kennedy P Kennedy D. Football supporters and the commercialisation of football: comparative responses across Europe. Soccer Soc. (2012) 13(3):327–40. 10.1080/14660970.2012.655503

[45] Nash R. English football fan groups in the 1990s: class, representation and fan power. Soccer Soc. (2001) 2:39–58. 10.1080/714866720

[46] Dubal S. The neoliberalization of football: rethinking neoliberalism through the commercialization of the beautiful game. Int Rev Soc Sport. (2010) 45(2):123–46. 10.1177/1012690210362426

[47] Strutner M Parrish C Nauright J. Making soccer “major league” in the USA and beyond: major league soccer’s first decade. Sport Hist Rev. (2014) 45:23–36. 10.1123/shr.2012-0017

[48] Allison R Barranco R. A rich white kid sport? Hometown socioeconomic, racial, and geographic composition among U.S. women’s Professional soccer players. Soccer Soc. (2021) 22(5):457–69. 10.1080/14660970.2020.1827231

[49] Pepple J. Soccer, the left, & the farce of multiculturalism. Bloomington, IN: AuthorHouse (2010).

[50] Pelak C. Negotiating gender/race/class constraints in the new South Africa: a case study of women’s Soccer. Int Rev Sociol Sport. (2005) 40(1):53–70. 10.1177/1012690205052165

[51] Parnell D Fitzpatrick D May A Widdop P. The political economy of grassroots football: from obscurity to austerity. In: Carr J, Parnell D, Widdop P, Power MJ, et al. editors. Football, politics and identity. London: Routledge (2021). p. 177–92.

[52] Friedkin NE. Social cohesion. Annu Rev Sociol. (2004) 30:409–25. 10.1146/annurev.soc.30.012703.110625

[53] Allport GW. The nature of prejudice. Oxford: Perseus Books Group (1954).

[54] Billings SB Chyn E Haggag K. The long-run effects of school racial diversity on political identity. Am Econ Rev. (2021) 3(3):267–84. 10.1257/aeri.20200336

[55] Krasnoff LS. Devolution of les bleus as a symbol of a multicultural French future. Soccer Soc. (2017) 18:311–9. 10.1080/14660970.2016.1166775

[56] Stehle M Weber BM. German Soccer, the 2010 World Cup, and multicultural belonging. Ger Stud Rev. (2013) 36(1):103–24. https://www.jstor.org/stable/43555294

[57] Williams J Peach J. We are all foxes now: sport, multiculturalism and business in the era of disneyization. Sport Soc. (2018) 21(3):415–33. 10.1080/17430437.2017.1346616

[58] Lowe M. Types of contact: a field experiment on collaborative and adversarial caste integration. Am Econ Rev. (2021) 111(6):1807–44. 10.1257/aer.20191780

[59] Cantillon Z. Urban reimaging, heritage and the making of a world-class city: the commonwealth walkway as mega-event legacy project. Herit Soc. (2022):1–18. 10.1080/2159032X.2022.2127177

[60] Kidd B. The culture wars of the Montreal Olympics. Int Rev Sociol Sport. (1992) 27(2):151–61. 10.1177/101269029202700205

[61] Zhou Y Ap J. Residents’ perceptions towards the impacts of the Beijing 2008 Olympic Games. J Travel Res. (2009) 48(1):78–91. 10.1177/0047287508328792

[62] Grix J. “Image” leveraging and sports mega-events: Germany and the 2006 FIFA World Cup. J Sport Tour. (2012) 17(4):289–312. 10.1080/14775085.2012.760934

[63] Black D van der Westhuizen J. The allure of global games for “semi-peripheral” polities and spaces: a research agenda. Third World Q. (2004) 25(7):1195–214. 10.1080/014365904200281221

[64] Dahl GB Kotsadam A Rooth DO. Does integration change gender attitudes? The effect of randomly assigning women to traditionally male teams. Q J Econ. (2021) 136(2):987–1030. 10.1093/qje/qjaa047

[65] Alesina A Tabellini M. The political effects of immigration: Culture or economics? (No. w30079). Cambridge, MA: National Bureau of Economic Research (2022).

[66] Nielsen. Women’s football report 2019 (2019). Available at: https://nielsensports.com/womens-football-2019/.

[67] Giuliano P Tabellini M. The seeds of ideology: Historical immigration and political preferences in the United States (No. w27238). Cambridge, MA: National Bureau of Economic Research (2020).

[68] Halla M Wagner A Zweimuller J. Immigration and voting for the far right. J Eur Econ Assoc. (2017) 15(6):1341–85. 10.1093/jeea/jvx003

[69] Dustmann C Vasiljeva K Damm AP. Refugee migration and electoral outcomes. Rev Econ Stud. (2019) 86(5):2035–91. 10.1093/restud/rdy047

[70] Campos C Hargreaves Heap S Leite Lopez de Leon F. The political influence of peer groups: experimental evidence in the classroom. Oxf Econ Pap. (2017) 69(4):963–85. 10.1093/oep/gpw065

[71] Harmon N Fisman R Kamenica E. Peer effects in legislative voting. Am Econ J: Appl Econ. (2019) 11(4):156–80. 10.1257/app.20180286

[72] Finan F Seira E Simpser A. Voting with one’s neighbors: evidence from migration within Mexico. J Public Econ. (2021) 202:104495. 10.1016/j.jpubeco.2021.104495

[73] Calderon A Fouka V Tabellini M. Racial diversity and racial policy preferences: the great migration and civil rights. Rev Econ Stud. (2022). 10.1016/j.jpubeco.2021.104495. [Epub ahead of print]36798741

[74] Angrist JD Pischke JS. Mostly harmless econometrics: An empiricist's companion. Princeton: Princeton University Press (2009).

[75] de Chaisemartin C D'Haultfœuille X. Two-way fixed effects estimators with heterogeneous treatment effects. Am Econ Rev. (2020) 110(9):2964–96. 10.1257/aer.20181169

[76] Jakiela P. Simple diagnostics for two-way fixed effects. Department of Economics Working Papers 2021-05, Williamstown: Department of Economics, Williams College (2021).

[77] Fair R. Econometrics and presidential elections. J Econ Perspect. (1996) 10(3):89–102. 10.1257/jep.10.3.89

[78] Kahane L. It’s the economy, and then some: modeling the presidential vote with state panel data. Public Choice. (2009) 139:343–56. 10.1007/s11127-009-9397-z

[79] Peterson J Smith K Hibbing J. Do people really become more conservative as they age? J Pol. (2021) 82(2):600–11. 10.1086/706889

[80] Rose AK Spiegel MM. The Olympic effect. Econ J. (2011) 121(553):652–77. 10.1111/j.1468-0297.2010.02407.x

[81] Reuters. Article (2022). Available at: https://www.reuters.com/world/us/us-supreme-court-overturns-abortion-rights-landmark-2022-06-24/.

